# Alterations in acid-base balance, blood gases, and hematobiochemical profiles of whole-blood and thoracic fluid in goats with contagious caprine pleuropneumonia

**DOI:** 10.14202/vetworld.2021.1874-1878

**Published:** 2021-07-20

**Authors:** Mohamed Tharwat

**Affiliations:** 1 Department of Veterinary Medicine, College of Agriculture and Veterinary Medicine, Qassim University, P.O. Box 6622, Buraidah, 51452, Saudi Arabia; 2 Department of Animal Medicine, Faculty of Veterinary Medicine, Zagazig University, 44519, Zagazig, Egypt

**Keywords:** acid-base balance, blood gases, contagious caprine pleuropneumonia, goat, *Mycoplasma*

## Abstract

**Background and Aim:**

Contagious caprine pleuropneumonia (CCPP) is a highly contagious and fatal disease affecting goats and some wild ruminants. It is a cause of major economic losses in the goat industry in Africa, Asia, and the Middle East. This study aimed to investigate the acid-base balance, blood gases, and hematobiochemical profiles of whole-blood and fluid collected from the thoracic cavity in goats with CCPP.

**Materials and Methods:**

Fifty-five goats suffering from weight loss, anorexia, dyspnea, polypnea, cough, and nasal discharges due to CCPP were studied. Twenty-five healthy goats were used as controls. Diseased animals were enrolled in this study based on a positive serological latex agglutination test (LAT) that confirmed the detection of *Mycoplasma capricolum* subsp. *capripneumoniae*. The control goats were enrolled based on a negative result of the LAT.

**Results:**

Compared with a mean value of 7.38±0.04 in controls, the pH in the diseased group was 7.41±0.05. The blood pressure of carbon dioxide (PCO_2_), pressure of oxygen (PO_2_), base excess (BE), bicarbonate (HCO_3_), total carbon dioxide (TCO_2_), and saturation of oxygen (SO_2_) were lower in goats with CCPP than in controls. However, the anion gap (AnGap) was higher in the diseased goats than in the healthy ones. Compared with the levels in blood samples, the thoracic fluid PCO_2_, PO_2_, BE, and SO_2_ were higher while pH, HCO_3_, TCO_2_, and AnGap were lower. Compared with the findings in healthy goats, hematological alterations included significant increases in white blood cells and neutrophils, and a significant decrease in the red blood cell count. In the thoracic fluid, neutrophilic leukocytosis was a remarkable finding. The serum concentrations of globulin, blood urea nitrogen, and glucose, and the activities of aspartate aminotransferase (AST) and g-glutamyl transpeptidase (GGT) increased significantly compared with those in controls. In contrast, serum concentrations of albumin, calcium, and magnesium, and the activity of alkaline phosphatase (ALP) decreased significantly compared with those of healthy animals. The activities of ALP, AST, GGT, and creatine kinase and the concentration of phosphorus were higher in thoracic fluid than the serum values in the diseased group.

**Conclusion:**

When compared with the healthy controls, goats with CCPP have metabolic acidosis. Compared with the levels in healthy goats, the blood PCO_2_, PO_2_, BE, HCO_3_, TCO_2_, and SO_2_ are low in goats with CCPP; however, the AnGap is higher in diseased goats.

## Introduction

Contagious caprine pleuropneumonia (CCPP) is a highly contagious severe mycoplasmal disease that affects the vast majority of goat populations and is caused by *Mycoplasma capricolum* subsp. *capripneumoniae* (*Mccp*). This disease rarely affects sheep and does not infect cows [1]. CCPP has been reported to have a wide distribution among sheep and goats covering diverse areas, especially in Africa and Asia [2]. This disease is economically devastating for the goat industry in Africa, Asia, and the Middle East [3-5]. Goats that are raised in over 40 countries globally are affected by CCPP, so it poses a serious threat to the goat industry worldwide [1].

CCPP mainly affects the respiratory system. It is characterized in its acute form by fever, anorexia, and severe respiratory symptoms including cough, nasal discharge, difficulty breathing, increased respiratory rate, and pleuropneumonia [6]. The diagnosis of CCPP in veterinary clinics depends on both clinical and postmortem examinations, which should be confirmed by laboratory tests [4]. Confirmation of *Mccp* is carried out by different methods, including microscopic examination of pulmonary exudation, histopathology of pulmonary parenchyma, molecular identification, and typing, namely, by polymerase chain reaction (PCR), detection of antigen in affected tissue by gel precipitin tests, and characterization by modern serological techniques [1]. The more recently developed methodologies include the latex agglutination test (LAT), competitive enzyme-linked immunosorbent assay, PCR, and restriction fragment length polymorphism [1,7-9]. Evaluation of cavity fluids including abdominal, thoracic, and pericardial effusions in the laboratory is beneficial for evaluating disease severity [10,11].

Grossly, consolidation of the lungs is the main finding in CCPP-affected goats, followed by alveolar exudation and pleural fluid accumulation and pleural adhesion, along with fibrinous pleuritis and peribronchial cuffing of inflammatory mononuclear cells in pulmonary tissue [2]. During the progression of CCPP, changes of various hematobiochemical variables have been reported in mycoplasma-affected goats compared with the findings in control ones. These changes included anemia, leukocytosis followed by leukopenia, hypoproteinemia, hypoalbuminemia, and elevated aspartate aminotransferase (AST), alanine aminotransferase, calcium, glucose, and globulin [1]. Therefore, it is expected that blood pH and blood gases will differ significantly compared with those of healthy goats, so it is vital to also treat the acid-base disturbances. It was recently reported that controlling the acid-base status will increase the value of therapies for many human diseases [12].

This investigation was designed to study the acid-base status and blood gases in goats with CCPP using whole-blood samples and also fluid collected from the thoracic cavity. In parallel with this, the hematobiochemical profiles were also investigated in both samples.

## Materials and Methods

### Ethical approval

The study design was approved by the Animal Ethical Committee, Deanship for Scientific Research, Qassim University, Saudi Arabia (approval number 2015-3436). The goats were maintained and treated according to the Laboratory Animal Control Guidelines of Qassim University, which basically conform to the Guide for the Care and Use of Laboratory Animals of the National Institutes of Health in the USA (NIH publications No. 86 to 23, revised 1996).

### Study period and location

The study was conducted from February 2010 to August 2015 at the Veterinary Teaching Hospital, Qassim University, Saudi Arabia.

### Experimental group

The experimental design has been reported previously [4]. Briefly, 55 goats aged 2.5±1.1 years and weighing 26.4±10.1 kg, which suffered from decreased body weight, complete loss of appetite, and respiratory symptoms including difficulty breathing, increased respiratory rate, coughing, and profound nasal execrations were examined. All of the goats with CCPP had positive LAT results [4].

### Control group

Healthy goats (n=25, age 2.8±0.9 years; weight 31.0±12.7 kg) were used as a control group. All of the healthy animals had a negative LAT result [4].

### Blood sampling and owners’ agreement

From each goat, a 7 mL blood sample was collected: 2 mL in an ethylenediaminetetraacetic acid (EDTA) tube and 5 mL in a heparinized tube. The diseased goats in this study were included in the investigation after the provision of informed agreement from their owners, and permission to euthanize and examine the goats at necropsy was given.

### Sampling of thoracic fluid

The selected area for collecting pleural fluid was aseptically prepared, after which it was injected with lidocaine hydrochloride (5 mL of 2%) as a local anesthetic. First, a small skin incision was made with a scalpel point, followed by ultrasound-guided insertion of an 18 g×80 mm needle into the pleural cavity. Seven milliliters of thoracic fluid were then withdrawn from the diseased group and placed in EDTA (2 mL) and heparin tubes (5 mL). Unfortunately, no fluid was imaged in the control goats, so no pleural fluid was collected from the controls to be analyzed for comparison with data from sick goats.

### Acid-base and blood gas analyses

A portable clinical veterinary analyzer (I-STAT^®^, Abaxis, California, USA) was used to evaluate the acid-base status and blood gases in the heparinized blood samples. The pH, pressure of oxygen and carbon dioxide (PO_2_ and PCO_2_, respectively), bicarbonate (HCO_3_), base excess (BE), total carbon dioxide (TCO_2_), saturation of oxygen (SO_2_), AnGap, sodium, and chloride were measured immediately to stop changes in these variables [13]. Measurements of acid-base and blood gas analyses were carried out in 38 samples of whole blood, 15 thoracic fluid samples, and 15 samples of whole blood in the control goats.

### Determination of hematological and biochemical parameters

Total leukocytic count and its subsets erythrocyte count, hematocrit (HCT), and hemoglobin were determined for the EDTA samples using VetScan HM5 (Abaxis, CA, USA). For the heparinized blood samples, an automated biochemical analyzer was used to measure the concentrations of blood urea nitrogen (BUN), calcium, phosphorus, magnesium, glucose, total protein (TP), albumin, and globulin (VetScan VS2, Abaxis, California, USA). The serum activities of AST, g-glutamyl transferase (GGT), alkaline phosphatase (ALP), and creatine kinase (CK) were also determined by VetScan VS2. Determination of hematological and biochemical parameters was carried out in 38 samples of whole blood, 15 thoracic samples, and 15 samples of whole blood in the control goats.

### Statistical analysis

Data were assessed for normality using the D’Agostino Pearson test. The data were presented as means±standard deviations since no significant deviation from normality was observed. Data were analyzed statistically using the Statistical Package for Social Sciences program version 25.0, (IBM, Chicago, USA) [14]. A paired t-test for repeated samples was used to compare the acid-base status, blood gases, and hematobiochemical variables between goats with CCPP and the healthy ones. The significance was set at p≤0.05.

## Results

Compared with the levels in the heparin blood samples, the thoracic fluid PCO_2_, PO_2_, BE, and SO_2_ levels were high while pH, HCO_3_, TCO_2_, and AnGap were low. Table-1 displays the means±SD and the 25%, 50%, 75%, and 95% percentiles of acid-base balance and blood gases of blood samples collected from goats with CCPP versus healthy control goats. Compared with a mean value of 7.38±0.04 in controls, the pH in the diseased group was 7.41±0.05, with no significant difference between them (p=0.2). Compared with the values in healthy animals, the blood PCO_2_, PO_2_, BE, HCO_3_, TCO_2_, and SO_2_ were lower in diseased goats (p<0.05). However, the AnGap was higher in the diseased goats than in healthy ones (p<0.05).

**Table-1:** Acid-base balance and blood gases in goats with contagious caprine pleuropneumonia versus healthy controls.

Parameters	Whole blood (n=38)					Controls (n=15)					p-value
	
	Mean±SD	Percentiles (%)				Mean±SD	Percentiles (%)				
	
		25	50	75	95		25	50	75	95	
pH	7.41±0.05	7.39	7.43	7.44	7.45	7.38±0.04	7.38	7.41	7.45	7.52	0.2
PCO_2_ (mmHg)	29.8±5.6	24.9	28.4	33.9	38.6	39.9±6.3	35.6	37.9	43.8	50.6	<0.0001
PO_2_ (mmHg)	28±4	25	27	33	34	38±6.4	35	36	43	48	0.002
BE (mmol/L)	−5.9±4.8	−7.3	−5.5	−4.0	1.5	1.9±4.7	0.0	3.0	4.0	8.2	<0.0001
HCO_3_ (mmol/L)	18.8±4.2	17.0	19.3	20.5	25.4	27.4±4	25.2	27.3	29.1	32.4	<0.0001
TCO_2_ (mmol/L)	19.7±4.4	18.0	19.5	21.8	25.0	27.5±4.1	25.3	28.0	29.8	33.6	0.0001
SO_2_ (%)	55±8	49	53	61	65	65.3±6.6	64	65	69	73	0.0006
AnGap (mmol/L)	21.0±3.2	18.3	20.5	22.8	25.3	4.1±0.5	3.9	4.1	4.3	4.7	0.0001

PCO_2_=Partial pressure of carbon dioxide, PO_2_=Partial pressure of oxygen, BE=Base excess, HCO_3_=Bicarbonate, TCO_2_=Total carbon dioxide, SO_2_=Oxygen saturation, AnGap=Anion gap

Neutrophilic leukocytosis was a remarkable finding in the thoracic fluid. The hematological profiles in goats with CCPP compared with those of control animals are shown in Table-2. Compared with controls, hematological alterations included significant elevations in white blood cells (WBCs) and neutrophils (p<0.05). In contrast, the red blood cell (RBC) count in affected goats decreased significantly compared with that in healthy goats (p=0.0005). Other hematological measurements including lymphocytes, monocytes, hemoglobin, and HCT did not differ significantly when compared with those of controls (p>0.05).

**Table-2:** Hematological parameters in goats with contagious caprine pleuropneumonia versus healthy goats.

Parameters	Whole blood (n=38)					Controls (n=15)					p-value
	
	Mean±SD	Percentiles (%)				Mean±SD	Percentiles (%)				
	
		25	50	75	95		25	50	75	95	
WBCs (×10^9^/L)	20.4±15.2	9.1	15.7	30.3	50.2	14.6±3.6	12.3	15.3	16.9	18.8	0.008
LYM (×10^9^/L)	7.3±6.9	2.3	4.5	9.4	22.1	6.2±3.2	4.8	5.6	6.5	10.0	0.4
MON (×10^9^/L)	0.5±1.0	0.1	0.2	0.3	2.8	0.9±3.9	0.1	0.1	0.1	0.2	0.7
NEU (×10^9^/L)	12.7±10.9	4.7	8.0	22.0	33.3	9.3±4.1	6.5	8.7	11.0	17.4	0.008
RBCs (×10^12^/L)	14.1±5.1	10.2	13.9	17.8	21.5	16.6±2.1	14.9	16.5	17.6	20.0	0.0005
HB (g/dL)	13.0±4.1	8.1	9.9	11.2	13.7	12.0±2.7	9.5	10.6	11.5	12.2	0.03
HCT (%)	24.7±8.2	20.6	25.6	27.0	35.1	26.1±3.2	23.3	26.2	28.7	29.8	0.2

WBCs=White blood cells, LYM=Lymphocyte, MON=Monocyte, NEU=Neutrophil, RBCs=Red blood cells, HB=Hemoglobin, HCT=Hematocrit

The activities of ALP, AST, GGT, and CK and the concentration of phosphorus were higher in thoracic fluid than those in serum in diseased goats. Table-3 shows different biochemical parameters in goats with CCPP compared with those of healthy control animals. The serum levels of globulin, BUN, and glucose, and the activities of AST and GGT increased significantly compared with those of controls (p<0.05). On the other hand, the serum levels of albumin, calcium, and magnesium, and the activity of ALP decreased significantly compared with those of healthy goats (p<0.05). Other biochemical variables including the serum concentrations of TP and phosphorus, and the activity of CK did not differ significantly compared with the controls (p=0.1).

**Table-3:** Biochemical parameters in goats with contagious caprine pleuropneumonia versus healthy control goats.

Parameters	Whole blood (n=38)					Controls (n=15)					p-value
	
	Mean±SD	Percentiles (%)				Mean±SD	Percentiles (%)				
	
		25	50	75	95		25	50	75	95	
ALB (G/L)	33.5±7.4	26.8	32.5	40.5	43.3	44.8±2.3	43.0	44.0	47.0	48	<0.0001
ALP (U/L)	34±25	21	28	34	76	85±27	59	83	105	128	<0.0001
AST (U/L)	155±160	83	107	147	306	73±20	54	67	94	101	0.03
CA (MMOL/L)	1.9±0.5	1.9	2.0	2.2	2.5	2.3±0.2	2.2	2.4	2.5	2.6	0.003
GGT (U/L)	67±36	47	54	74	113	42±7	37	42	45	52	0.003
TP (G/L)	68.7±9.4	61.8	71.0	74.0	82.5	72.3±6	69	73	76	79	0.1
GLOB (G/L)	35.6±9.7	29.8	34.5	39.8	51.9	27.9±5.0	25	29	30	35	0.004
BUN (MG/dL)	11.2±11.8	4.25	6.5	14.0	36.0	3.6±1.7	2.5	3.4	4.2	5.7	0.008
CK (U/L)	378±649	152	173	312	109	177±59	128	155	227	264	0.1
PHOS (MMOL/)	1.7±0.6	1.4	1.7	2.0	2.4	2.1±0.8	1.2	2.2	2.8	3.0	0.1
MG (MMOL/L)	0.7±0.3	0.6	0.7	0.9	1.1	1.2±0.5	0.8	1.0	1.5	1.8	0.001
GLU (MG/dL)	116±30	95	109	142	154	78±26	62.5	68.5	89.5	113.5	0.005

TP=Total protein, ALB=Albumin, ALP=Alkaline phosphatase, AST=Aspartate aminotransferase, CA=Calcium, GGT=γ-glutamyl transferase, GLOB=Globulin, BUN=Blood urea nitrogen, CK=Creatine kinase, PHOS=Phosphorus, MG=Magnesium, GLU=Glucose

## Discussion

In the present study, thoracic fluid samples were difficult to be collected from the control goats for comparisons with the diseased group. Normally, a small quantity of fluid is found in the thoracic, pericardial, and abdominal cavities, providing lubrication and preventing friction between adjacent organs and the body cavity walls [15]. Effusions of these cavities refer to the excessive accumulation of fluids in these cavities, which are prevalent in veterinary medicine [16]. The etiology of cavity fluid accumulation includes cardiovascular disorders, neoplasia, trauma, and hypoalbuminemia. Suitable collection and appropriate evaluation of body cavity fluids can supply the practitioners with valuable information that assists in recognizing the disease process that causes fluid accumulation [10]. In goats with advanced CCPP, profuse straw-colored fluid accumulates in the pleural cavity [17-19].

In the host organism, homeostasis is stimulated as a result of any bacterial infection in different ways based on the pathogen itself, site of infection, pathology, and severity of infection [20]. In the current study, the acid-base parameters were investigated in goats with CCPP due to *Mccp* infection and, although the difference was not significant, the blood pH in goats with CCPP was higher than that in controls. This increase could be easily explained by the increase in respiratory rate and loss of CO_2_ during expiration. The significant decreases of blood PCO_2_, TCO_2_, HCO_3_, and BE also support this explanation. The BE constitutes HCO_3_ and other basic elements, and therefore, BE is considered a more sensitive indicator of metabolic acidosis than only HCO_3_ [21]. In this study, the low values of HCO_3_, BE, and TCO_2_ may be justified due to the metabolic acidosis, which is the cause of why BE mean values were negative. Another indicator of the resulting metabolic acidosis in goats with CCPP in this study was the significant difference in the level of AnGap [22].

Concerning the hematobiochemical parameters in goats with CCPP, neutrophilic leukocytosis, and decreased RBC count were the outstanding significant findings. Interestingly, the numbers of WBCs and neutrophils were also high in thoracic fluid. The results of leukocytosis and anemia agree well with those obtained by Abdelsalam *et al*. [23] and Mondal *et al*. [24]. Usually, in field cases, hematological findings are not so relevant to the diagnosis of CCPP [1]. Similar findings of increased globulin, glucose, and AST and decreased albumin have also been reported by Mondal *et al*. [24]. In this study, the reason why the activities of ALP, AST, GGT, and CK were higher in thoracic fluid than in serum may be the severe pulmonary damage.

## Conclusion

It is concluded from this study that goats with CCPP have metabolic acidosis when compared with healthy controls. Compared with the values in healthy goats, the blood levels of PCO_2_, PO_2_, BE, HCO_3_, TCO_2_, and SO_2_ were low in goats with CCPP; however, the AnGap was higher in the diseased goats. A limitation of this study was the failure to collect thoracic samples from the healthy group for comparisons with diseased goats. A future study is, therefore, warranted to collect thoracic samples from healthy goats at a slaughterhouse for comparisons with thoracic samples of goats affected by CCPP.

## Author’s Contributions

MT: Designed and performed the experiment, analyzed the data, wrote and revised the manuscript, and prepared the tables. The author has read and approved the final manuscript.
